# 3D Rigid Registration of Intraoperative Ultrasound and Preoperative MR Brain Images Based on Hyperechogenic Structures

**DOI:** 10.1155/2012/531319

**Published:** 2012-01-19

**Authors:** Pierrick Coupé, Pierre Hellier, Xavier Morandi, Christian Barillot

**Affiliations:** ^1^LaBRI CNRS, UMR 5800, Université Bordeaux, 33405 Talence Cedex, France; ^2^University of Rennes I, CNRS UMR 6074, IRISA, 35042 Rennes, France; ^3^INRIA, VisAGeS U746 Unit/Project, IRISA, 35042 Rennes, France; ^4^INSERM, VisAGeS U746 Unit/Project, IRISA, 35042 Rennes, France; ^5^Technicolor Corporate Research Rennes Laboratory, 1 Avenue de Belle Fontaine, CS 17616, 65576 Cesson-Sévigné Cedex, France; ^6^University Hospital of Rennes, 35043 Rennes, France

## Abstract

The registration of intraoperative ultrasound (US) images with preoperative magnetic resonance (MR) images is a challenging problem due to the difference of information contained in each image modality. To overcome this difficulty, we introduce a new probabilistic function based on the matching of cerebral hyperechogenic structures. In brain imaging, these structures are the liquid interfaces such as the cerebral falx and the sulci, and the lesions when the corresponding tissue is hyperechogenic. The registration procedure is achieved by maximizing the joint probability for a voxel to be included in hyperechogenic structures in both modalities. Experiments were carried out on real datasets acquired during neurosurgical procedures. The proposed validation framework is based on (i) visual assessment, (ii) manual expert estimations , and (iii) a robustness study. Results show that the proposed method (i) is visually efficient, (ii) produces no statistically different registration accuracy compared to manual-based expert registration, and (iii) converges robustly. Finally, the computation time required by our method is compatible with intraoperative use.

## 1. Introduction

Due to its low cost, its real-time imaging capabilities, and its noninvasive nature, ultrasound (US) imaging has become a popular modality. These attributes have been used for a large number of clinical applications. In neurosurgery, ultrasound imaging has been employed in many cases of brain examinations over the last two decades [1]. Several studies demonstrated that ultrasonography can be used for locating tumors, defining their margins, differentiating their internal characteristics, and for detecting of brain shift and residual tumoral tissues [2]. At present, 3D US imaging is integrated within the neuronavigation systems to provide a useful and efficient intraoperative tool [3]. Ultrasound imaging has also been shown to be a promising method for quantifying and for correcting brain shift in Image-Guided Neurosurgery (IGNS) [4–14].

During a neurosurgical procedure, the ultrasound probe is tracked by the neuronavigation system which computes the 3D positions and orientations of the B-scans. Matching between the intraoperative US images and the preoperative MR image is ensured by a rigid registration. In phantom [6] and animal studies [11], the matching accuracy between intraoperative B-scans and preoperative images has been quantified between 1.5 mm and 3 mm. Nevertheless, in clinical context, the matching error can reach 10 mm (see Table 1). This error includes tool calibration errors (the position localizer and the US probe), tool localization errors (tracking system error), and registration errors from the neuronavigation system. 

Registration approaches based on classical image similarity measures such as the Sum Square Difference (SSD), Mutual Information (MI), or Correlation Ratio (CR) are known to fail to robustly register MR and US images [15]. Therefore, other options have been investigated.

- Landmark-based registration represents the majority of the approaches [6, 7, 9, 13, 14, 16]. The motivation is bound to the difficulty of finding a function matching US image intensities with MR image intensities. These methods are based on the matching of manually defined points [7], lines representing the vascular system [6, 13, 14, 16], or cortical surface [9].
- Intensity-based approaches using histogram-based similarity measures tend to overcome the problem by preprocessing the images in order to register more similar images [4, 17].
- By introducing the Bivariate Correlation Ratio (BCR), Roche et al. [15] incorporated the transformation of MR to pseudo-US image as a function into the similarity measure.

In this paper, we propose a new objective function based on the matching of the cerebral hyperechogenic structures such as sulci and the cerebral falx, and the lesion when the corresponding tissue is hyperechogenic. The registration is achieved by maximizing the correlation value between the US image and the probabilistic map of hyperechogenic structures estimated from MR image. The proposed method is thus a compromise between landmark and intensity-based approaches.

- As with landmark-based approaches, only regions considered as relevant are used to drive the registration procedure. In our method, these regions are the hyperechogenic structures of the brain.
- As with intensity-based methods, the proposed approach does not require segmentation of the US image which is a challenging problem.

## 2. Materials and Methods

### 2.1. Method Overview

**Figure 1:**
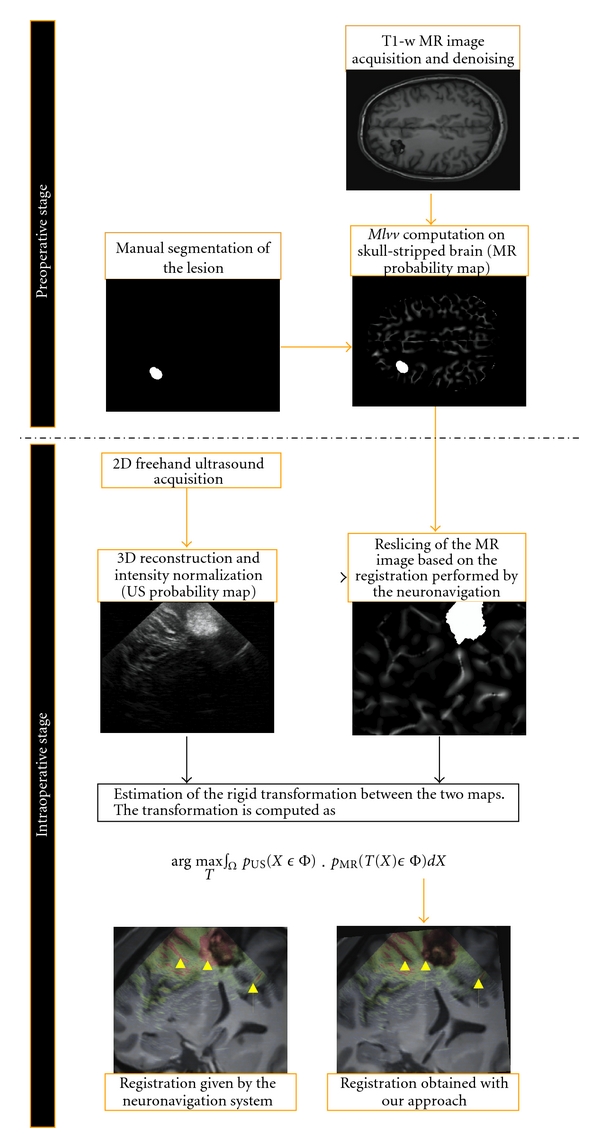
Illustration of the performed workflow to achieve the registration. The skull stripping, the denoising, the *MLvv* computation, and the segmentation of lesion are performed before the neurosurgical procedure. The 3D reconstruction of intraoperative volume, the reslicing of the MR map, and the estimation of the transformation are then performed during the neurosurgical procedure.

The scheme of the overall workflow is presented in Figure 1. First, the “hyperechogenic” structures present in MR image (i.e., the structures visible in MR image expected to be hyperechogenic in intraoperative US) are detected with the *MLvv* operator [18, 19]. In brain imaging, these structures are the liquid interfaces such as the cerebral falx and the sulci, in addition to the lesions when the corresponding tissue is hyperechogenic (e.g., cavernoma or glioma). The curvature-based *MLvv* operator was first introduced in [18, 19] before being used to detect the sulci and the cerebral falx in [20–22]. The US image and the probability map of the hyperechogenic structures extracted from MR image are then registered by maximizing the probability for a voxel to be included in hyperechogenic structures in both modalities.

Contrary to histogram-based approaches that match all the information in both images, the proposed approach consists of matching only hyperechogenic structures [23], which makes it more robust to artefacts such as acoustic shadows. Indeed, in US imaging, the bright areas provide information on the underlying structures whereas the dark areas can correspond to the underlying anatomical structure or acoustic shadows [24]. Moreover, the accuracy of sulci matching is an important issue since these structures are used by the neurosurgeon during the neurosurgical procedure [25]. Finally, by using the natural property of US imaging to detect the hyperechogenic structures, the method does not require segmentation of the US image. This way, the method is less sensitive to error of US image segmentation and is less time consuming during the intraoperative stage.

### 2.2. Probabilistic Objective Function

The proposed registration process is based on the estimation of the transformation $\hat{T}$ maximizing the joint probability for a voxel *X* = (*x*, *y*, *z*) to be included in hyperechogenic structures in both modalities:

(1) $$\begin{array}{c} \hat{T}=\arg\underset{T}{\max}\int_{\Omega }p\left(X\in \Phi_{\text{US}},T\left(X\right)\in \Phi_{\text{MR}}\right)dX, \end{array}$$

where *p*(*X* ∈ Φ_US_) is the probability for *X* to be included in an hyperechogenic structure from the US image and *p*(*X* ∈ Φ_MR_) is the probability for *X* to be included in an hyperechogenic structure (in the sense of the ultrasound image) from the T1-w MR image. Assuming that the probabilities are independent, we can write

(2) $$\begin{array}{c} \hat{T}=\arg\underset{T}{\max}\int_{\Omega }p\left(X\in \Phi_{\text{US}}\right)\cdot p\left(T\left(X\right)\in \Phi_{\text{MR}}\right)dX. \end{array}$$

Our objective function can be viewed as the maximization of the correlation value between the two probability maps of hyperechogenic structures extracted from both modalities.

### 2.3. Construction of the Probability Maps

In order to construct the probability maps, we define a function *f* matching the intensity of both the US image and the MR image with the probability for *X* to be included in hyperechogenic structures:

(3) $$\begin{array}{c} p\left(X\in \Phi \right)=f\left(u\left(X\right)\right), \end{array}$$

where *u* : *Ω* ↦ ℝ is an image defined on *Ω*.

#### 2.3.1. Intraoperative US Image

For the intraoperative US image *U*, by definition *f* is the identity function:

(4) $$\begin{array}{c} p\left(X\in \Phi_{\text{US}}\right)=U\left(X\right). \end{array}$$

The intensity of *U* is only scaled between 0 and 1 during surgery to fit with our probabilistic framework.

#### 2.3.2. Preoperative MR Image

For the preoperative MR image *V*, the evaluation of *f* is done prior to surgery and is based on both the detection of the liquid interfaces with the *MLvv* operator and the segmentation of the pathological tissues.

The *Lvv* operator is a robust intensity-based curvature detector [18] based on the first and second derivatives of the image intensities. The first and second derivatives are combined to obtain an operator less sensitive to flat areas with low gradients. This kind of operator is used to detect ridge-like features in images, with negative value for crests in the intensity domain and positive value for valleys in the intensity domain. In [19], the *MLvv* has been proposed for multimodal registration of CT and MR images. In our case, as in [20–22], the *MLvv* is used to extract the hyperechogenic structures (sulci, cerebral falx) from T1-w MR image. Overall, curvature information has been used by several other authors to characterize cortical features [26–28]. Most of these methods are based on geodesic curvature computed on cortical surfaces.

**Figure 2:**
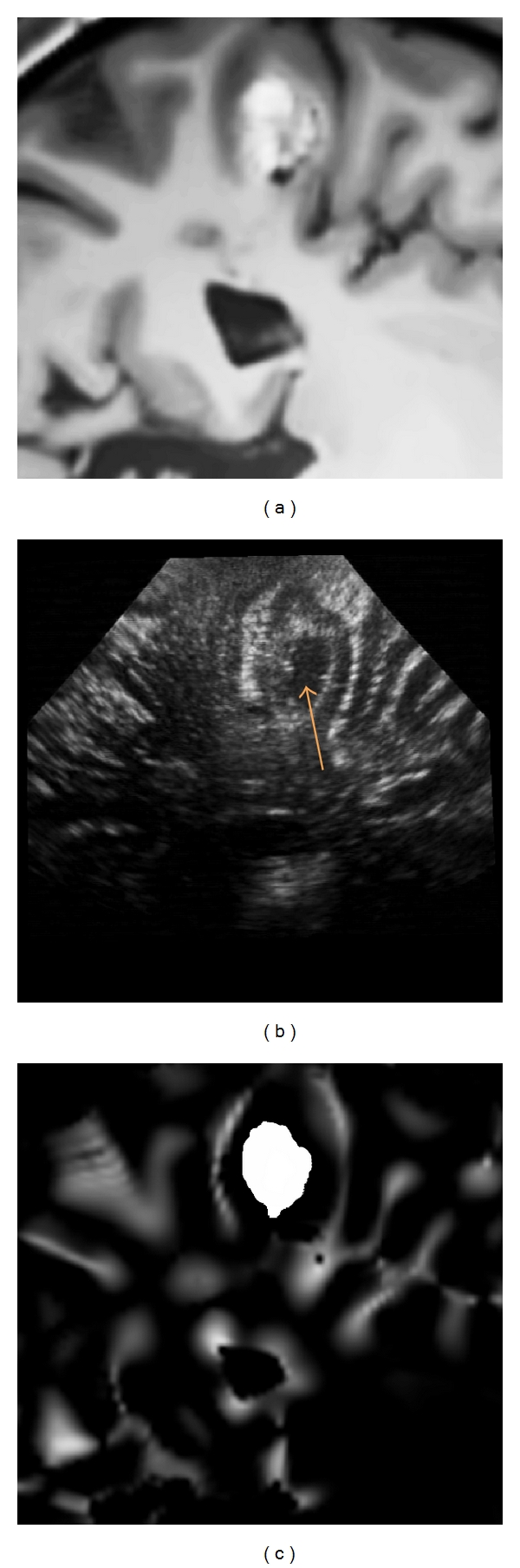
Patient 1. (a) The denoised MR image corresponding to US image (b). (c) The probability map based on *MLvv* operator and extracted from denoising MR image. The preoperative MR image is resliced with the registration matrix provided by the neuronavigation system. The matching is not perfect due to the initial error, estimated around 4.2 mm by the experts for this patient. As visible on the US image, the lesion presents a hypoechogenic central area (indicated on the US image by a narrow).

**Figure 3:**
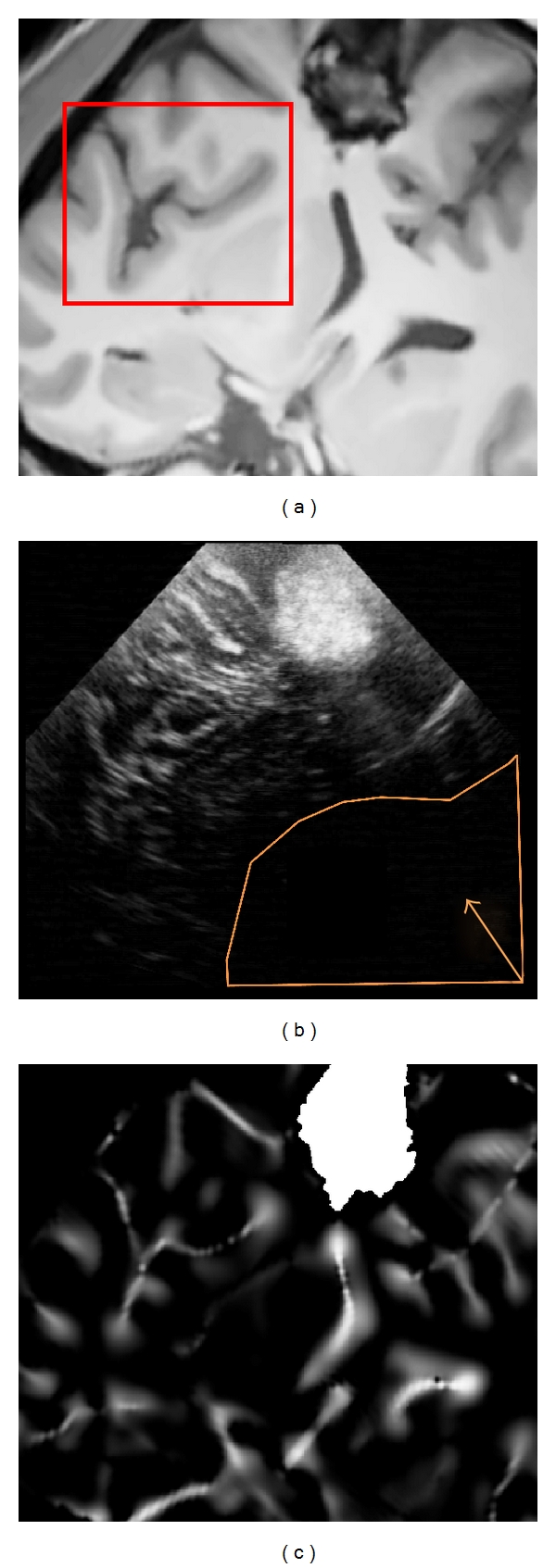
Patient 2. (a) The denoised MR image corresponding to US image (b). (c) The probability map based on *MLvv* operator and extracted from denoising MR image. The preoperative MR image is resliced with the registration matrix provided by the neuronavigation system. The matching is not perfect due to the initial error, estimated around 8.5 mm by the experts for this patient. The red box shows a sulcal area where the matching between US image and the *MLvv*-based probability map is visually high. A large acoustic shadow is visible in US around the ventricle area (indicated in orange on the US image).

**Figure 4:**
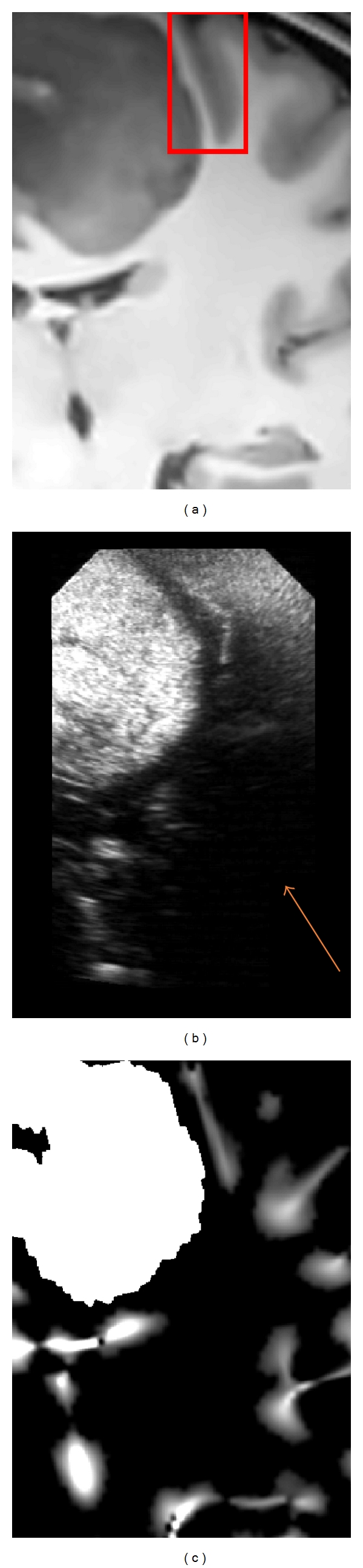
Patient 3. (a) The denoised MR image corresponding to US image (b). (c) The probability map based on *MLvv* operator and extracted from denoising MR image. The initial error is estimated around 5 mm by the experts for this patient. In the red box, the *MLvv* operator efficiently detects the sulci also visible in the US image. This case presents a limited field of view while the lesion is large. Moreover, an acoustic shadow is visible on the right lower part of the US image (indicated on the US image by a narrow).

In T1-w MR images, the sulci are valleys (negative ridges) in the intensity domain. By using the positive values of *MLvv*, the sulci and the cerebral falx can be efficiently detected [20–22]. Figures 2, 3, and 4 show the positive values of *MLvv* operator.

Finally, our function *f* is defined as

(5) $$\begin{array}{c} p\left(X\in \Phi_{\text{MR}}\right)=MLvv\left(V\left(X\right)\right){𝕀}_{M1}\left(X\right)+\Psi \left(X\right){𝕀}_{M2}\left(X\right), \end{array}$$

where *𝕀*_*M*_ is the indicator function for the set *M*:

- *M*_1_ = {*X* ∈ *Ω*, such  that  *MLvv*(*V*(*X*))⩾0},
- *M*_2_ = {*X* ∈ *Ω*, such  that  *X*  belongs  to the lesional tissue}.

As for the US intensities, the positive values of the *MLvv* are scaled between 0 and 1. The *MLvv* operator is defined in 3D as

(6) $$\begin{array}{cc} & MLvv\left(V\left(x,y,z\right)\right)\\ & \;\;=-\frac{1}{2{||\vec{w}||}^{2}}[{\frac{\partial V\left(X\right)}{\partial x}}^{2}\left(\frac{\partial^{2}V\left(X\right)}{\partial y^{2}}+\frac{\partial^{2}V\left(X\right)}{\partial z^{2}}\right)\\ & \;\;\;\;\;\;\;\;\;-2\frac{\partial V\left(X\right)}{\partial y}\frac{\partial V\left(X\right)}{\partial z}\frac{\partial^{2}V\left(X\right)}{\partial y\partial z}\\ & \;\;\;\;\;\;\;\;\;+{\frac{\partial V\left(X\right)}{\partial y}}^{2}\left(\frac{\partial^{2}V\left(X\right)}{\partial x^{2}}+\frac{\partial^{2}V\left(X\right)}{\partial z^{2}}\right)\\ & \;\;\;\;\;\;\;\;\;-2\frac{\partial V\left(X\right)}{\partial x}\frac{\partial V\left(X\right)}{\partial z}\frac{\partial^{2}V\left(X\right)}{\partial x\partial z}\\ & \;\;\;\;\;\;\;\;\;+{\frac{\partial V\left(X\right)}{\partial z}}^{2}\left(\frac{\partial^{2}V\left(X\right)}{\partial x^{2}}+\frac{\partial^{2}V\left(X\right)}{\partial y^{2}}\right)\\ & \;\;\;\;\;\;\;\;\;-2\frac{\partial V\left(X\right)}{\partial x}\frac{\partial V\left(X\right)}{\partial y}\frac{\partial^{2}V\left(X\right)}{\partial x\partial y}], \end{array}$$

where $||\vec{w}{||}^{2}=\partial V{\left(X\right)}^{2}/\partial x+\partial V{\left(X\right)}^{2}/\partial y+\partial V{\left(X\right)}^{2}/\partial z$. Ψ(*X*) is the probability given to *X* in the segmentation of pathological tissue *M*_2_. Ψ is used to incorporate *a priori* on pathology. For pathological tissue such as cavernoma or low-grade glioma, Ψ(*X*) is high since these tissues are hyperechogenic.

### 2.4. Preprocessing of the MR Data before Surgery

First, skull stripping is performed from the T1-w MRI sequence [29]. We choose to remove the skull prior to *MLvv* computation because this structure does not appear in the area of the craniotomy. The raw MR images are then denoised using an optimized Non-Local Means filter (https://www.irisa.fr/visages/benchmarks/) [30] before applying the *MLvv* operator to the brain tissues. The use of a denoising stage makes the computation of the *MLvv* more stable. Indeed, the presence of noise may create false positive or negative curvatures which could bias the registration framework. After applying the *MLvv* operator, only the positive values (i.e., the sulci and the falx) are kept in the processing stream. Finally, the *MLvv* map and the segmentation *M*_2_ are merged together (see Figures 2, 3, and 4). In our experiments, the segmentation of pathology was manually performed by the neuroanatomist before the surgical procedure (see Figure 1). The computational time required by preprocessing steps performed during preoperative stage was 4 minutes for skull stripping and 3 minutes for denoising on a Pentium M 2 GHz. In addition, 3–8 minutes were required for manual segmentation of the lesion according to its size on a Stealth Station TREON (Medtronic Inc., Minneapolis, USA). Since these steps are performed before surgery, there is no impact on practical value of the proposed method.

### 2.5. Data Acquisition

T1-w SENSE 3D sequences were used to acquire preoperative T1-weighted MR images on a 3T Philips Gyroscan scanner (Best, the Netherlands). During the neurosurgical procedure, the US probe (Sonosite Inc. Bothell, WA. USA, cranial 7–4 MHz probe) was tracked by the Polaris cameras of the Stealth Station TREON (Medtronic Inc., Minneapolis, USA). The SonoNav software of the neuronavigation system was used to acquire the 2D B-scans and the probe positions. From the 2D B-scans and their positions, a 3D volume was reconstructed with the Probe Trajectory method [31]. The experiments were carried out on 3 patients. For each patient, a sequence of images was acquired before opening the dura. Some studies have considered quantitative measurement of brain shift during surgical procedures and showed that nonsignificant displacement occurred before dura opening [32, 33]. Thus, we assumed that the transformation between intraoperative US and preoperative MR was rigid. The characteristics of reconstructed volumes are

- for patient 1 a 3D volume of 486 × 462 × 206 voxels with a resolution of 0.15 × 0.14 × 0.14 mm^3^,
- for patient 2 a 3D volume of 510 × 423 × 174 voxels with a resolution of 0.21 × 0.19 × 0.20 mm^3^,
- for patient 3 a 3D volume of 265 × 450 × 324 voxels with a resolution of 0.19 × 0.17 × 0.18 mm^3^.

### 2.6. MR-US Registration of the Neuronavigation System

During all the neurosurgical procedure, the coordinate system of the preoperative MR image and the coordinate system of the intraoperative field are related by a rigid registration. The rigid registration of the neuronavigation system is based on surface matching between the preoperative MR image and the position of points acquired on the patient's head with the position localizer. First, the skin is extracted from the MR image by manual thresholding. A cloud of points is then continuously acquired on the patient's head close to the eyes region by moving the position localizer. Following this, one point is acquired on each ear with another point on the extremity of the patient's nose. Finally, the neuronavigation system performs a points to surface matching.

According to phantom and animal studies, the errors in probe calibration, 3D localization of the probe, and rigid registration performed by the neuronavigation system lead to a global error less than 3 mm [6, 10, 11]. The error due to the 3D localization of the probe is estimated to 0.35 mm for each marker on a tool from the manufacturer [34]. The error due to the calibration is generally estimated around 1.5 mm [6, 11]. In our case, the probe was calibrated with a Z-wire phantom by the manufacturer. Finally, the error due to rigid registration performed by the neuronavigation system has been estimated to be around 1.5 mm in [11].

### 2.7. Pathology of the Patients

**Figure 5:**
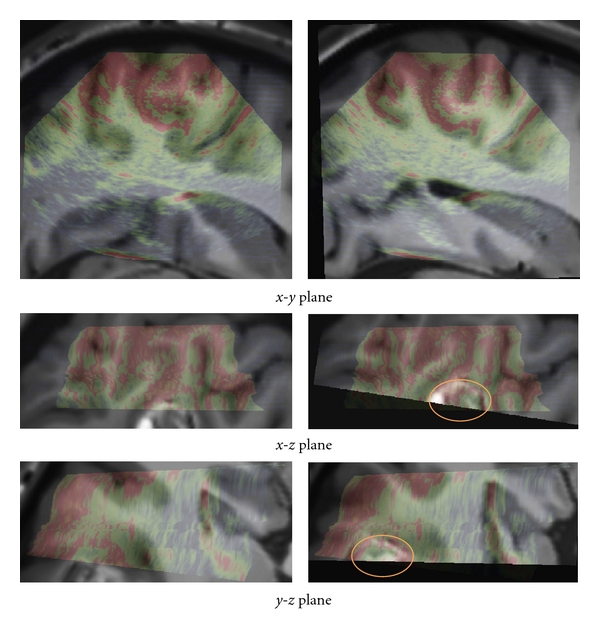
Patient 1. Left: registration given by the neuronavigation system. Right: the result after correction with our registration approach. The low intensities of US images are in green and the high in red. For this case, even if the lesion was not entirely included in the US volume, the proposed registration procedure converged. The location of the lesion is indicated by ellipses on the fused image after registration.

**Figure 6:**
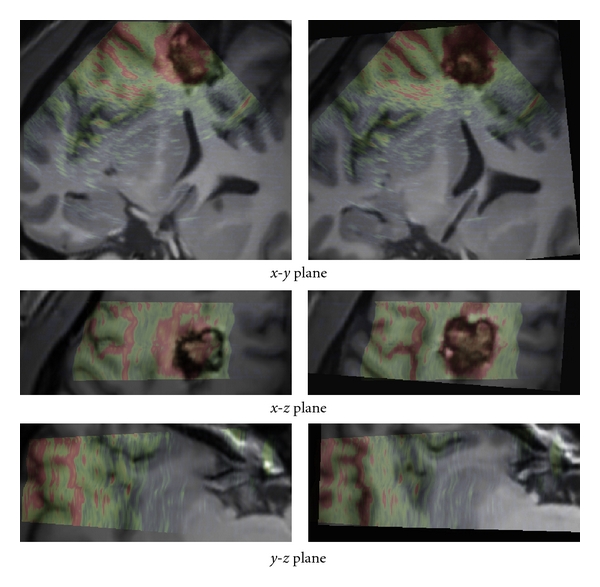
Patient 2. Left: registration given by the neuronavigation system. Right: results after correction with our registration approach. The low intensities of US images are in green and the high in red. In this case, the acoustic shadow artifact was present on the US image. The signal below the lesion was totally dark as shown in Figure 3. The proposed approach allowed to overcome these artifacts without specific detection of the shadows.

**Figure 7:**
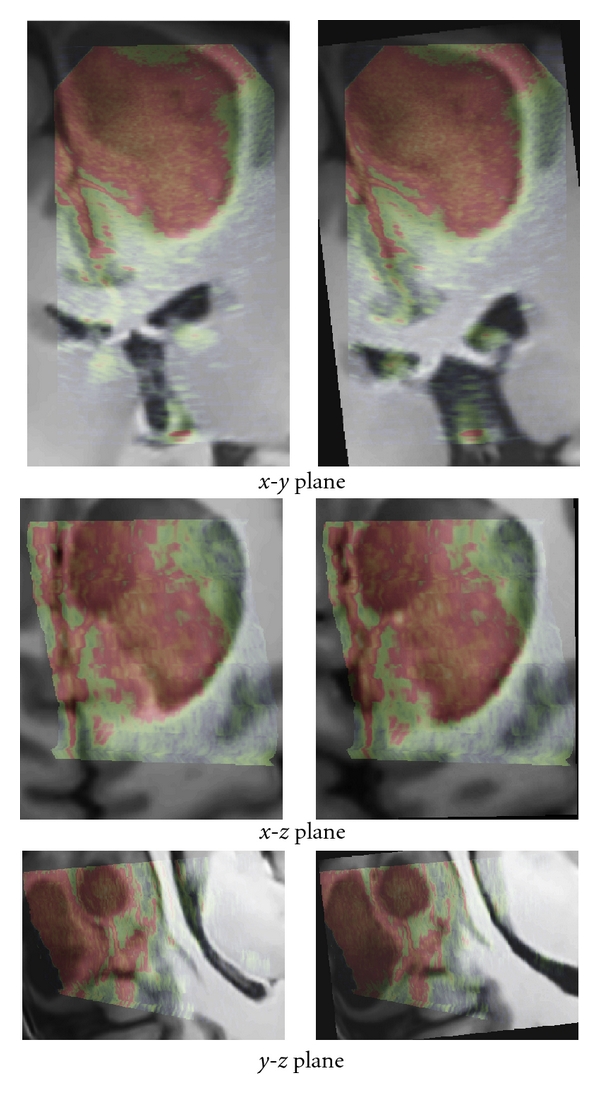
Patient 3. Left: registration given by the neuronavigation system. Right: the result after correction with our registration approach. The low intensities of US images are in green and the high in red.

In this study, hyperechogenic pathologies such as cavernoma (patient 1, see Figure 5 and patient 2, see Figure 6) and low-grade glioma (patient 3, see Figure 7) were considered. In T1-w MR images, the central part of cavernoma is usually heterogeneous (hyper- and hyposignal) and the outlying area appears in hyposignal. The low-grade gliomas are more homogeneous and appear in hyposignal in T1-w MR images. In US images, numerous studies showed that all solid brain tumors, metastatic brain lesions, and cavernomas exhibited echogenicity [35–39]. For brain gliomas, the higher its grade (more malignant), the more echogenic it is in US and the less homogeneous it appears. In our study, the corresponding lesional tissues were considered both homogeneous and hyperechogenic in US images. As such, Ψ(*X*) was set to 1 for all segmentation of pathological tissue *M*_2_ (see (5)). Typical examples of intraoperative images and probability maps are presented in Figures 2, 3, and 4.

### 2.8. Parameter Settings

The maximization of the joint probability (see (2)) is performed within a multiresolution procedure using the simplex algorithm [40]. During the experiments, the parameters of the simplex algorithm were tolerance = 0.1, stepsize = 1.5, and maximum number of iterations = 100. The coarsest resolution corresponded to the original volumes downsampled by a factor 3 and the finest resolution was that of the original volumes. The registration procedures take less than two minutes on Intel Pentium M at 2 GHz.

As with most derivative-based operators, the *MLvv* operator uses Gaussian kernel to compute the image derivatives. In [18], the authors showed that the convolution of the image with a derivative Gaussian kernel provides a well-posed approach of the differentiation problem. The standard deviation *σ* of the Gaussian kernel is called the image scale. This parameter has been shown as very stable for MR image sulci segmentation on numerous works [20–22], and thus, no tuning has been done for this parameter throughout our study. In our experiments, an image scale of 2 voxels has been used to compute the *MLvv* values. This value is consistent with other works [20–22] conducted on brain cortical segmentation where the scale parameter was always kept in this range.

### 2.9. Evaluation Framework

In order to evaluate our method, a validation framework with different approaches is proposed.

- First, a visual assessment is proposed.
- Second, a manual validation by experts is presented. This validation is divided in two parts: a point-based estimation of the rigid registration by 3 experts for the 3 patients and an evaluation of the residual error by all experts for 1 patient (postregistration error).
- Third, a study on convergence robustness was carried out.

The expert manual validation was difficult due to the time required. For each expert, 4 hours were required to perform the *a priori* estimation of the transformation for 3 patients.

#### 2.9.1. Visual Assessment

The visual assessment remains a valuable indicator of the registration accuracy. In [41], the observer discernibility of registration errors has been estimated around 0.2 mm. A study on visual inspection for image registration assessment can be found in [42]. In our paper, we propose an overlay of US and MR images before and after registration to assess the registration accuracy.

#### 2.9.2. Validation by Experts

First, the experts manually evaluate the rigid transformation between the intraoperative US and the MR image resliced with the rigid transformation given by the neuronavigation system. This estimation is denoted as *a priori* estimation of the registration. From this *a priori* estimation, the initial error (i.e., after the registration performed by the neuronavigation system) and the Target Registration Error (TRE) can be computed. The *a priori* estimation of the registration is used to show that there are no statistical differences between the expert-based transformations and the transformation estimated with our method in terms of the TRE.

The experts estimate the residual error after rigid registration based on a given transformation (either by our method or the point-based expert registrations). This estimation is called *a posteriori* evaluation of the residual error and is designed to show that experts do not detect significant differences when they inspect the registered volumes with our method or with their own manually defined transformations.

**A Priori Estimation of the Registration**

**Point Picking**

The *a priori* estimation of the registration is based on the location of ten points in the US image and the ten corresponding points in the MR image: each expert defines a set of point in the 3D reconstruction of the intraoperative ultrasound and its corresponding landmark in the resliced MR image. The resliced MR image is obtained with the rigid registration given by the neuronavigation system and has the same resolutions, dimensions, and field of view as the reconstructed US image. During the experiments, the experts used three orthogonal 2D views to define homologous points in the 3D volumes. For each volume, the visualization software was run independently, with the cursors in the two volumes unlinked. Each expert was allowed to choose their set of homologous points.

**Initial Error**

The initial error is computed by using the mean Euclidean distance between the homologous points defined by the experts in both modalities. The three samples (one per expert) containing the ten error values (one per point) are compared by using a Kruskal-Wallis test.

**Target Registration Error**

A leave-one-out procedure is used to compute the TRE of each point (i.e., Euclidean distance between homologous point after rigid transformation). First, one of the ten homologous points is removed from the set of points. The nine remaining homologous points are then used to compute a rigid transformation in the least squares sense. Finally, this rigid transformation is used to compute the TRE of the initially removed point. This procedure is repeated for all the ten points. The final TRE is the mean TRE over all the points. For each patient, the expert-based TRE and the TRE obtained with our method are compared by using a nonparametric Kruskal-Wallis test.

**A Posteriori Evaluation of the Residual Error**

**Point Picking**

First, the patient images are registered using several transformations. These transformations are (i) the three expert-based transformations (${\tilde{T}}_{1},{\tilde{T}}_{2},{\tilde{T}}_{3}$), (ii) the rigid transformation obtained with our method ($\hat{T}$), and (iii) the transformation computed using all the thirty points defined by the three experts ${\tilde{T}}_{\text{all}}$. The experts then define ten homologous points on the registered volumes. This procedure is performed for the five studied registrations on the patient 2 dataset. The positions of the points are fixed for all the experts.

**Residual Error**

As for initial error, the final error or residual error is simply obtained by computing the mean Euclidean distance between the homologous points defined by the experts in both modalities. The statistical comparison of the residual errors is performed on the five samples (one per transformation) containing ten errors values (one per point) with a Kruskal-Wallis test.

#### 2.9.3. Robustness Study

First, the US and resliced MR images of patient 2 are registered with the transformation ${\tilde{T}}_{\text{all}}$. Then, 100 rigid transformations are randomly generated with a translation along each axis uniformly distributed between 0 and 5 mm and with a rotation around each axis uniformly distributed between 0 and 5 degrees. Finally, each transformation is applied to the resliced MR image before performing registrations with the proposed method. The warping index *ω* [43] is used to compute the distance between the estimated transformation by the registration process $\hat{T}$ and the true transformation *T*:

(7) $$\begin{array}{c} \omega =\frac{1}{\left|\Omega \right|}\underset{X\in \Omega }{\sum }{||T^{-1}(X)-\hat{T}(X)||}_{2}, \end{array}$$

where ||·||_2_ is the *L*_2_-norm. The success rate is estimated by considering a success as a registration with a warping index inferior to 3.5 mm. This threshold has been chosen close to the upper bound of the TRE estimated by the experts (see distribution for patient 2 in Figure 9). Contrary to TRE estimated over selected points, the warping index is computed as the average error between the volumes over all the voxels.

## 3. Results

### 3.1. Visual Assessment

The registration results are first displayed for visual assessment. The results obtained with our method are presented in Figures 5, 6, and 7. For patient 1 (see Figure 5), even if the lesion was not entirely included in the US volume, the proposed registration procedure converged efficiently. For patient 2 (see Figure 6), acoustic shadows are present on the US image. The signal below the lesion tends to zero. The proposed approach overcomes these artifacts without specific detection of the shadows. For patient 3 (see Figure 7), despite the large size of the low-grade glioma and the limited field of view, our approach performed well.

### 3.2. Validation by Experts

#### 3.2.1. A Priori Estimation of the Registration

**Figure 8:**
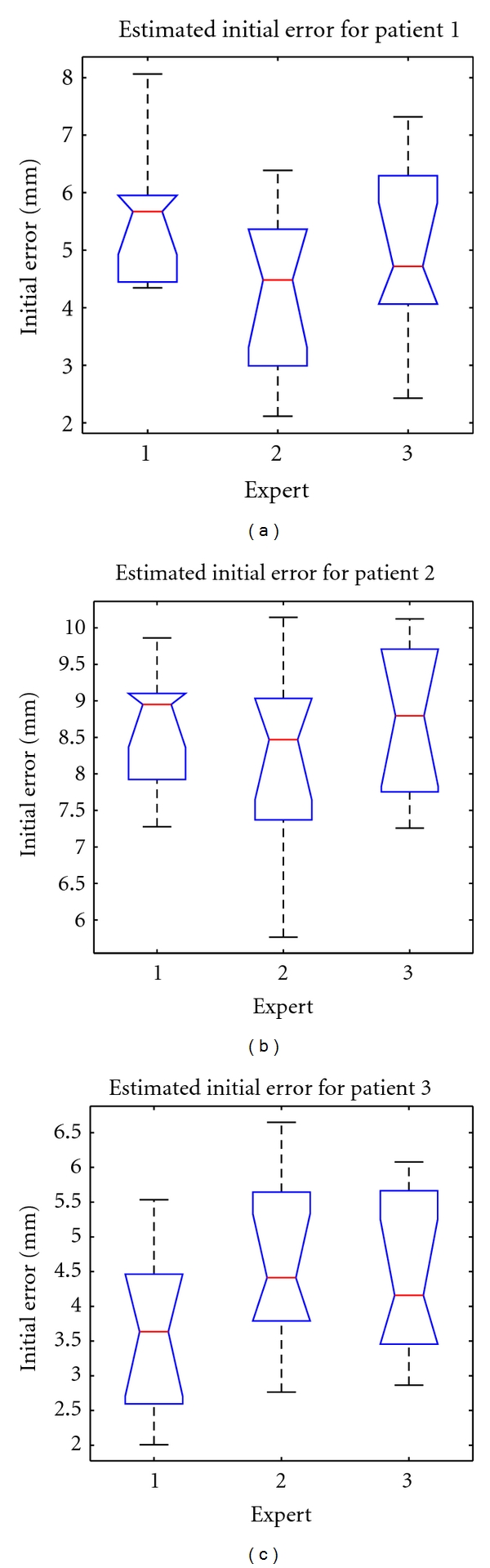
Initial errors (in mm) estimated by all experts for the each patient.

Table 1 presents the estimated initial error for the three patients by the three experts. The *P* value of the Kruskal-Wallis test showed that there was no significant difference between the expert estimations. Table 1 also shows the interindividual variability for the same measure between the experts.  Figure 8 summarizes the distribution of the error.

The estimated initial errors are significantly higher than values given in [6, 11] (<3 mm) or by the manufacturer (<1.5 mm). It is important to note that the ultrasound images used in our experiments were acquired in clinical context during a neurosurgical operation. The real neurosurgery context is likely more difficult than phantom and animal studies.

**Figure 9:**
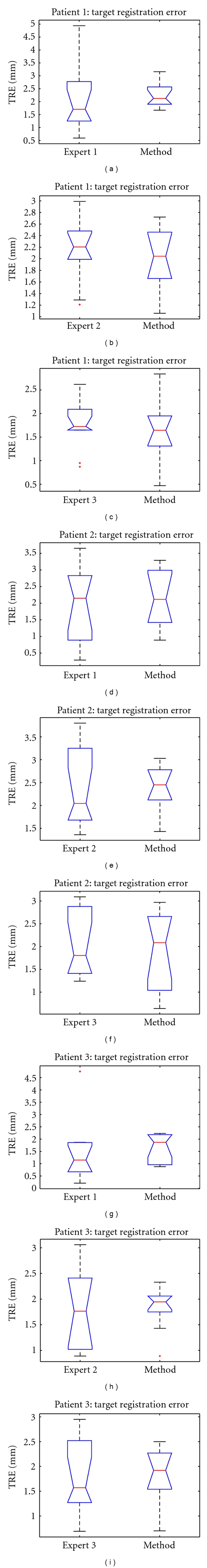
Target Registration Error (in mm) estimated by all experts for the each patient dataset.

Table 2 shows the TRE estimated by each expert, for each patient dataset. In all the cases, there were no statistically significant differences between the TRE obtained with expert-based estimations and the TRE obtained with our method. Figure 9 shows the result of the Kruskal-Wallis test. In all the cases, the experts and our method provided consistent results.

#### 3.2.2. A Posteriori Evaluation of the Residual Error

**Figure 10:**
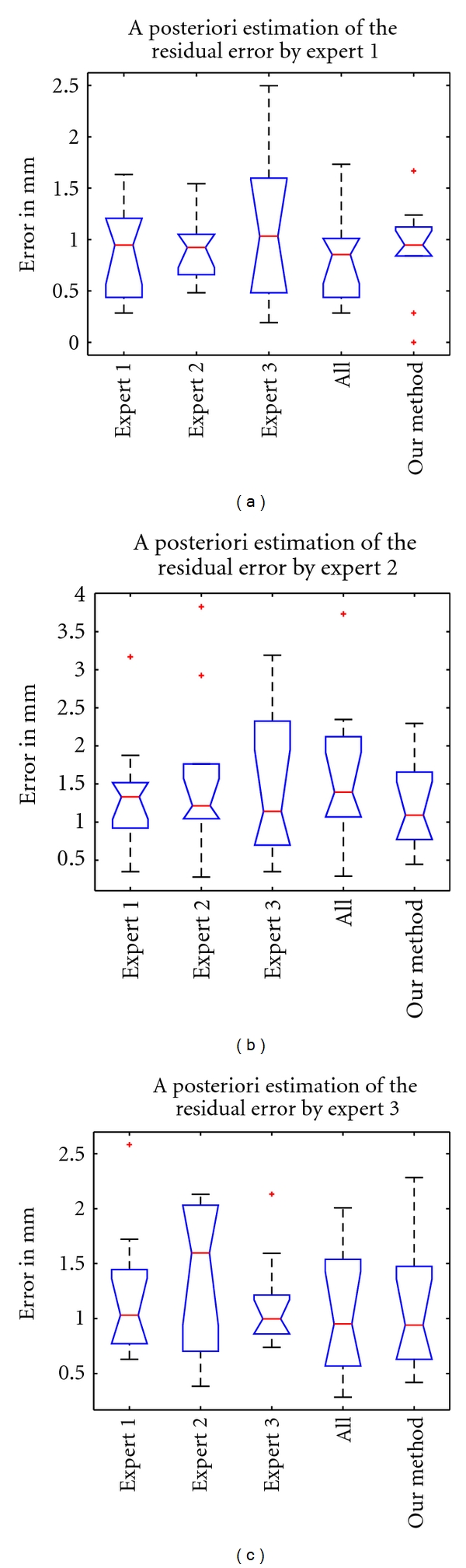
*A posteriori* residual error (in mm) estimated for all transformations by each expert.

Table 3 shows the expert-based estimation of the *a posteriori* residual error of the different registrations (manual-based $\tilde{T}$ and automatic $\hat{T}$) proposed for patient 2. The Kruskal-Wallis test shows that the errors associated with the transformations (${\tilde{T}}_{1},{\tilde{T}}_{2},{\tilde{T}}_{3},{\tilde{T}}_{\text{all}}$) and $\hat{T}$ are not significantly different.  Figure 10 shows the statistical distribution of the residual error for each transformation compared. Finally, the experts failed to detect significant differences between the manual-based registrations and our automatic registration. The residual error estimated by experts is around 1–1.5 mm for all the transformations.

### 3.3. Robustness Study

**Figure 11:**
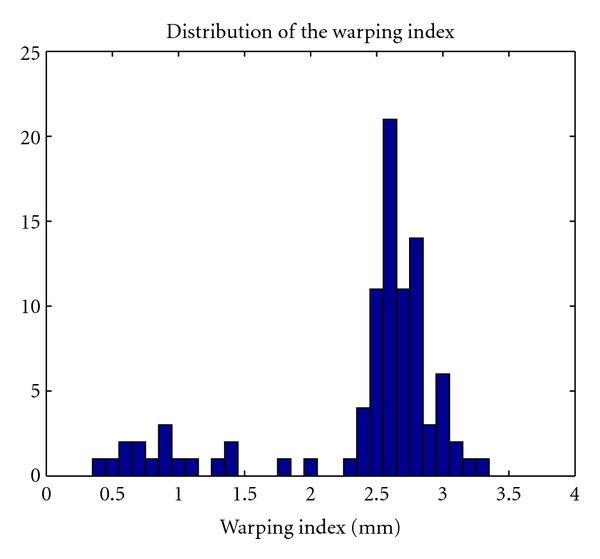
Distribution of the warping index in mm obtained with our method.

Table 4 shows the robustness and the warping index results obtained during the experiment. The proposed method obtained 92% of success rate with a mean warping index of 2.38 mm. This value is relative to the TRE of the used gold standard. Thus, it gives information about the distance between the transformation from all the experts (${\tilde{T}}_{\text{all}}$) and the final transformation provided by our method. This value is close to the TRE estimated for patient 2 in Table 2.  Figure 11 shows the distribution of the warping index. 

## 4. Discussion and Conclusion

This paper presents a new framework for the 3D rigid registration of US and T1-w MR brain images. In order to address this challenging problem, we propose an innovative probabilistic objective function that maximizes the joint probability of the (i) *a priori* most probable locations of hyperechogenic structure in the preoperative MR image and (ii) the highest intensities of the intraoperative US images. We show that the proposed method enables a robust registration of MR and US images in a computational time compatible with clinical use. All our experiments were carried out on real intraoperative data. The expert-based quantitative study shows that our method produces no statistically different registration compared to the *a priori* estimation of the registration by the experts. Moreover, the *a posteriori* estimation of the residual registration error shows that the experts failed to detect differences between manual registration and our automatic registration.

During our experiments, manual segmentation has been used to build the probability map. This segmentation is always available, since the neurosurgeon performed it before the surgery. In this paper, the used segmentations were the segmentations dedicated to the neurosurgery. However, the segmentation of the lesion could be automated [44, 45], and the different parts of pathologies (lesion, coagulated blood, cyst, necrotic tissue, etc.) could be defined. Through this, the simple model of homogeneous hyperechogenic lesion used in our experiment could be improved by using different hyperechogenic levels to the different pathological tissues. To evaluate the robustness of our method to heterogeneous lesion, more datasets are needed although this situation was present in case of patient 1.

The proposed method is related to the segmentation accuracy of the tumor in preoperative MR images. Although the segmentation of the MR image is not considered as difficult, this step may introduce some errors. Our experiments showed that the proposed method produced consistent results with manual segmentation used in clinical routine.

The presented clinical datasets showed that our method is robust to some discrepancies between the features present in both US and MRI probability maps. Based on the correlation of maps where only regions considered as relevant are used to drive the registration procedure, our method is able to deal with partially missing information resulting from a limited field of view or acoustic shadows. In case of patient 1 (see Figures 2 and 5), only a subpart of the lesion was visible in the reconstructed US image. In case of patient 2 (see Figures 3 and 6) the acoustic shadow below the lesion reduced information around ventricle in US. Moreover, the information derived from sulci was much more present in MR maps than in US map. Finally, in case of patient 3 (see Figures 4 and 7) the limited field of view and the large size of glioma reduced the importance of sulcal information. However, experiments using only the segmentation of the lesion or only sulcal information derived from *Mlvv* operator failed to provide satisfactory registration. This seems to indicate that a certain amount of homologous features has to be present in both probability maps to enable the method working. 

Finally, in our opinion, the proposed approach relies on a similar and complementary idea to the vessel-based method proposed by Reinertsen et al. [13, 14]. Indeed, in both cases, an implicit segmentation of salient features in US images (hyperechogenic structures in B-mode or vessels in Doppler) is matched with corresponding structures detected in MR images. Only the selected salient features differ between the methods. In [13, 14], the method utilizes vessels extracted from Doppler US images and their segmentation from MR images. Therefore, both methods have the advantage of not requiring segmentation of the US image and also being robust to US artefacts. However, the extraction of the vessel centerlines from MR images is a challenging problem and requires extensive processing.

Our method is dedicated to brain US imaging since *MLvv* operator is relevant for sulci and cerebral falx detection. As such, the application of the proposed framework to another body part requires adaptation of the hyperechogenic structure detection. Moreover, if T2-w MR image or another sequence is used as preoperative MR image, the selected values of the *MLvv* need to be adapted. Since the final aim of this US/MR registration method is to compensate for the brainshift, further works will investigate extension of our probabilistic objective function to non-rigid deformations.

## Figures and Tables

**Table 1:** Manual estimation of the initial error in mm (i.e., error of the registration given by the neuronavigation system).

*A priori* estimation of the registration: initial error in mm				
Mean (std)	Expert 1	Expert 2	Expert 3	*P* value
Patient 1	**5**.**52 **(1.15)	**4**.**31 **(1.55)	**5**.**00 **(1.50)	0.30
Patient 2	**8**.**64 **(0.89)	**8**.**31 **(1.24)	**8**.**76 **(1.04)	0.69
Patient 3	**3**.**56 **(1.09)	**4**.**61 **(1.39)	**4**.**38 **(1.13)	0.13

**Table 2:** Target registration error in mm. The *P* values correspond to Kruskal-Wallis test performed between the TRE obtained by experts and the TRE obtained with our method.

*A priori* estimation of the registration: target registration error in mm									
Mean (std)	Expert 1	Method	*P* value	Expert 2	Method	*P* value	Expert 3	Method	*P* value
Patient 1	2.26 (1.54)	2.25 (0.48)	0.28	2.16 (0.58)	2.03 (0.53)	0.50	1.75 (0.54)	1.63 (0.58)	0.50
Patient 2	1.90 (1.14)	2.11 (0.86)	0.68	2.39 (0.92)	2.39 (0.50)	0.76	2.02 (0.72)	1.89 (0.84)	0.71
Patient 3	1.47 (1.28)	1.64 (0.59)	0.20	1.82 (0.73)	1.84 (0.42)	0.97	1.78 (0.76)	1.79 (0.64)	0.88

**Table 3:** *A posteriori* evaluation of the residual error in mm in patient 2 by all experts. Our automatic registration $\hat{T}$ obtains no statistically different result compared to transformations extracted from experts estimation.

*A posteriori* evaluation of the residual error in mm						
Mean (std)	${\tilde{T}}_{1}$	${\tilde{T}}_{2}$	${\tilde{T}}_{3}$	${\tilde{T}}_{\text{all}}$	$\hat{T}$	*P* value
Expert 1	0.90 (0.47)	0.90 (0.31)	1.14 (0.74)	0.82 (0.45)	0.90 (0.43)	0.88
Expert 2	1.36 (0.79)	1.54 (1.07)	1.48 (1.00)	1.61 (0.98)	1.24 (0.60)	0.95
Expert 3	1.21 (1.27)	1.43 (0.65)	1.13 (0.44)	1.04 (0.56)	1.08 (0.57)	0.58

**Table 4:** Robustness study. Results of the proposed method for patient 2.

Success rate in %	*ω* in mm (Mean (std))
92	2.38 (0.71)
